# Supplementary material for: The Response of the Honey Bee Gut Microbiota to Nosema ceranae Is Modulated by the Probiotic Pediococcus acidilactici and the Neonicotinoid Thiamethoxam
Source: Microorganisms. 2024 Jan 18;12(1):192. doi: 10.3390/microorganisms12010192 (PMC10819737; doi:10.3390/microorganisms12010192)
Supplement: Supplementary file 1 [file microorganisms-12-00192-s001.zip › Figure S1.pdf]

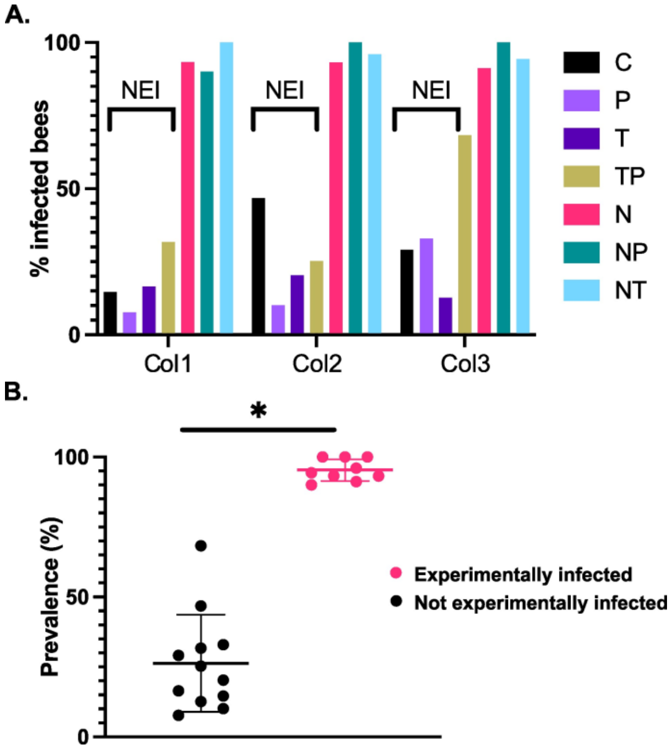

Figure S1. Prevalence of *Nosema ceranae* with or without experimental infection (**A**) Black and pink points represent all the replicates in not experimentally infected (NEI) n=12 and experimentally infected conditions n=9 (all groups included) respectively. Mann-Whitney U test, p-value < 0.0001 (**B**) Prevalence of *N. ceranae* per colony and per treatments; Control (C), *P. acidilactici* (P), Thiamethoxam (T), Thiamethoxam/*P. acidilactici* (TP), *N. ceranae* (N), *N. ceranae*/*P. acidilactici* (NP), *N. ceranae*/Thiamethoxam (NT)
